# Supplementary figures and images for: Dynamics of amylopectin granule accumulation during the course of chronic Toxoplasma infection is linked to intra-cyst bradyzoite replication
Source: mSphere. 2025 Jun 10;10(7):e00205-25. doi: 10.1128/msphere.00205-25 (PMC12306163; doi:10.1128/msphere.00205-25)

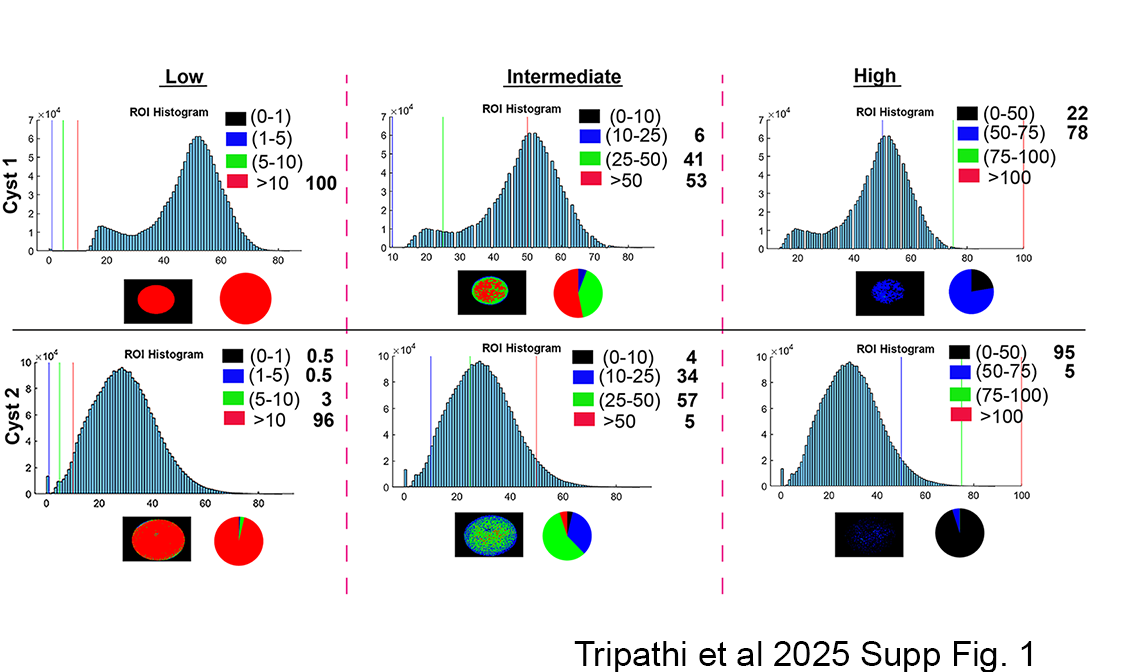

Supplement: Fig. S1 — Effect of threshold bins on PAs intensity distribution. [file msphere.00205-25-s0001.tif]

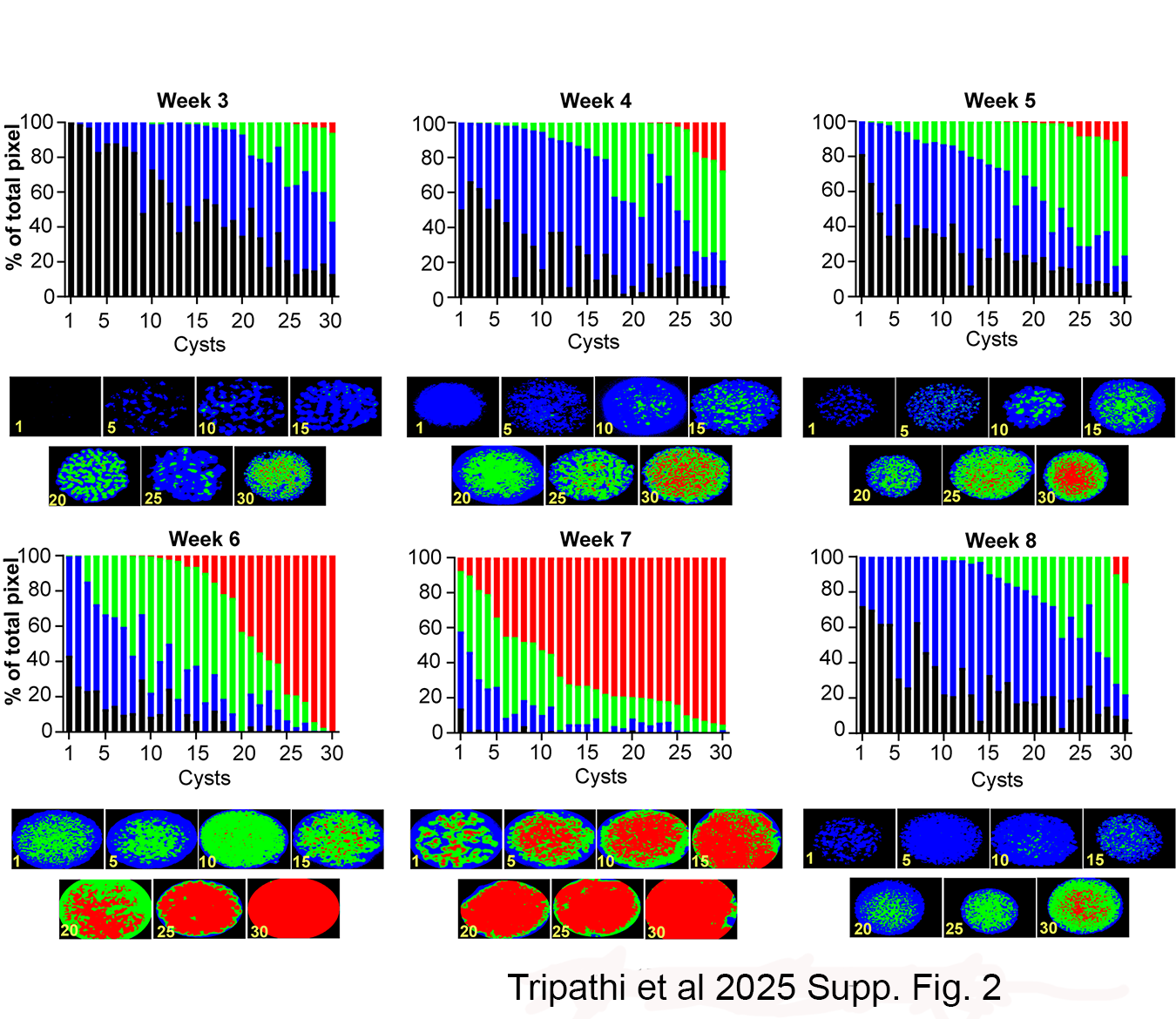

Supplement: Fig. S2 — PAS labeling of ethanol-fixed tissue cysts. [file msphere.00205-25-s0002.tif]

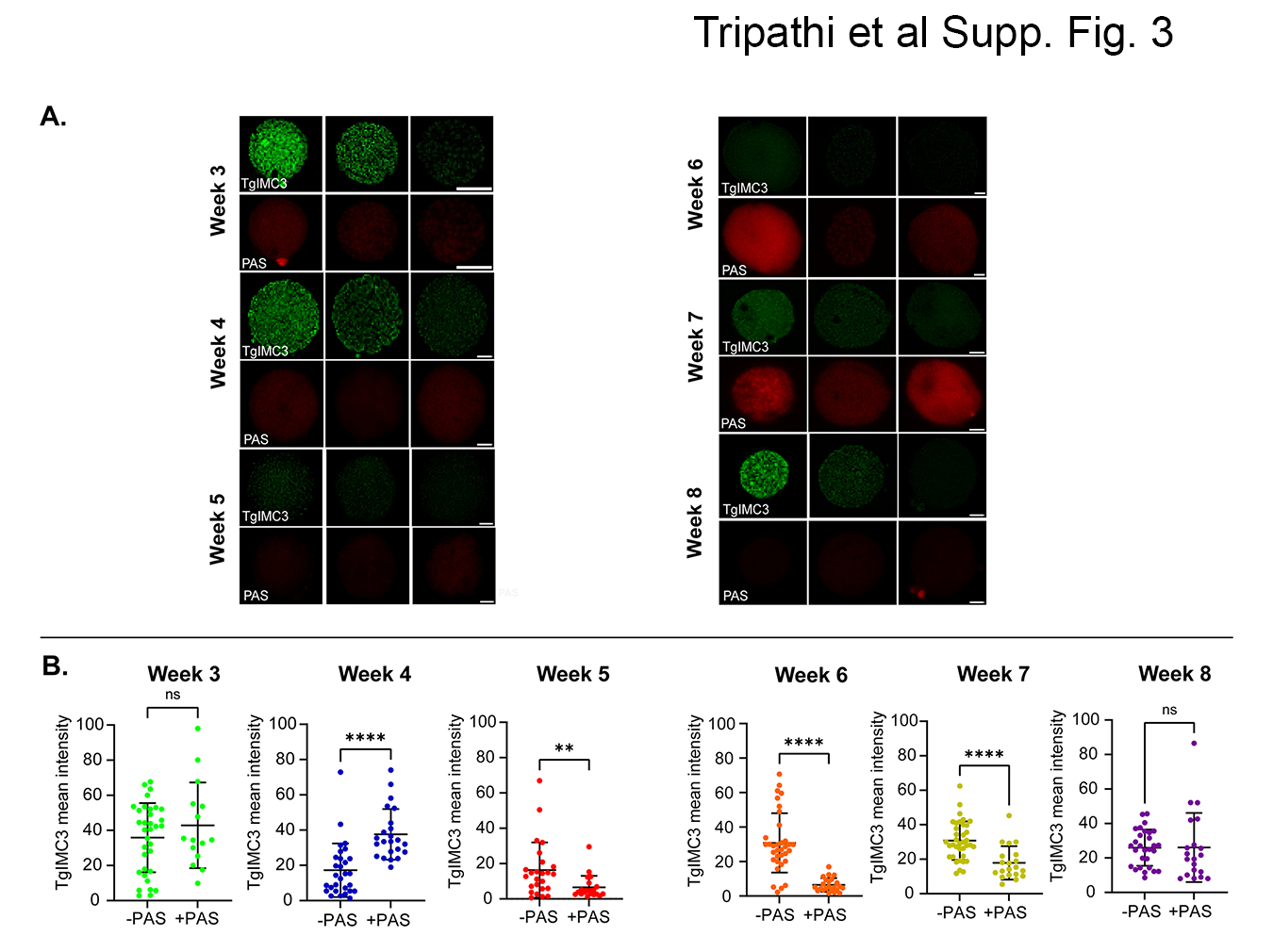

Supplement: Fig. S3 — PAS interference with TgIMC3 staining. [file msphere.00205-25-s0003.tif]

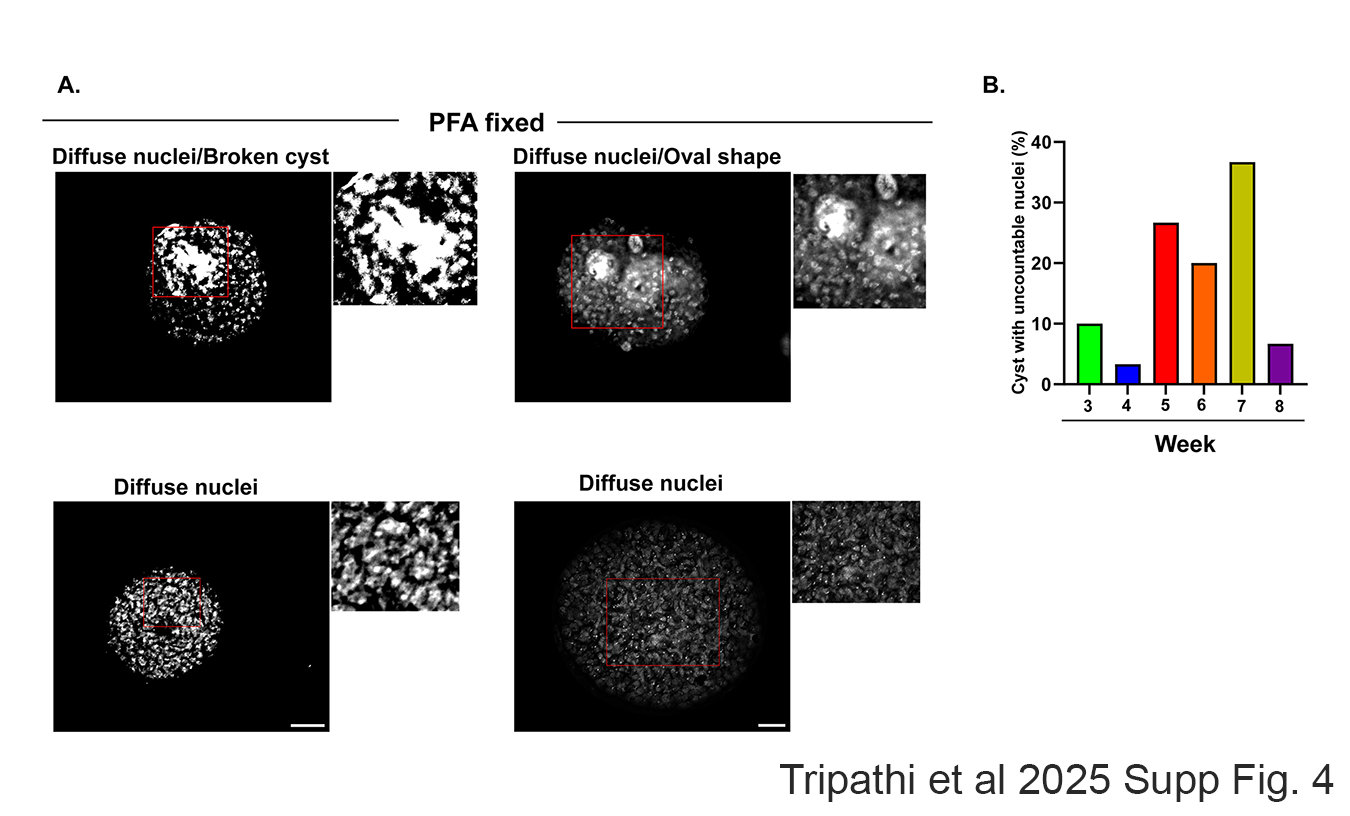

Supplement: Fig. S4 — PAS interference with DNA staining. [file msphere.00205-25-s0004.tif]
