# Supplementary material for: Dynamics of amylopectin granule accumulation during the course of chronic Toxoplasma infection is linked to intra-cyst bradyzoite replication
Source: mSphere. 2025 Jun 10;10(7):e00205-25. doi: 10.1128/msphere.00205-25 (PMC12306163; doi:10.1128/msphere.00205-25)
Supplement: Legends — for supplemental figures. [file msphere.00205-25-s0005.docx]

**Supplemental Data Figure Legends**

**Supplemental Figure S1. Effect of AmyloQuant threshold value settings on the distribution of PAS intensities.** Application of distinct threshold values in AmyloQuant in the same tissue cyst image (original images are adjusted) results in a fundamentally different patterns to define the background, low, intermediate and high bins thus impacting the representation of AG distribution. The optimized setting (Intermediate) of the threshold value provided the most balanced representation for typical tissue cysts. Thumbnails below the intensity histograms present the AmyloQuant generated heat maps for each of the threshold setting presented here.

**Supplemental Figure S2. Amylopectin dynamics revealed in methanol fixed tissue cyst following PAS staining and analysis in** **AmyloQuant** analysis of tissue cysts fixed in methanol. The bar graphs represent the ordered distribution of intensities in the background, low, intermediate and high ranges following the setting of bins at : BG: 0-10 (black), Low10-25 (blue), Intermediate 25-50 (green) and high >50 (red). The heatmaps under each set of bar graphs represent AmyloQuant generated spatial distributions of the 30 tissue cysts at 5 cyst intervals. While the overall AG pattern from week 3-8 is identical to that observed for PAS labeling of over this temporal course, a significant loss of signal is evident for methanol fixed tissue cysts. The amylopectin distribution pattern in chronic infection's early and late phases is similar to the PFA fixed and unaffected, irrespective of the fixation condition.

**Supplemental Figure S3. Interference of PAS staining with TgIMC3 labeling. (A)** Counter staining of PAS stained tissue cysts affected the efficiency of TgIMC3 labeling. In general poor TgIMC3 staining was noted in tissue cysts with high PAS labeling. Scale bar represents 10 μm. (**B**) Head to head comparison of TgIMC3 labeling on both untreated (-PAS) and PAS treated (+PAS) tissue cysts shows no difference at time points with low AG (week 3 and 8), but significant PAS dependent differences in TgIMC3 labeling at time points associated with higher levels of AG and thus higher PAS binding. Statistical Analysis: One way ANOVA with Mann-Whitney Test. P values: ns: not significant, **: 0.0047, ****= < 0.0001.

**Supplemental Figure S4. PAS staining impacts the quality of DAPI labeling compromising the ability to accurately count nuclei**. (**A**) PAS labeling of PFA and methanol (not shown) fixed tissue cysts affected the integrity and quality of nuclear staining that resulted in differentially diffuse nuclear profiles. While not uniform, the effect on PAS on nuclear staining was markedly more pronounced in tissue cysts from time points associated with higher levels of AG and thus PAS labeling. Additional factors leading to the exclusion of tissue cysts for analysis included non circular cysts and clearly damaged broken cysts. These issues precluded the accurate demarcation of the whole tissue cyst in AmyloQuant affecting the accuracy of both PAS and nuclear quantification. (**B**) The proportion of PAS/DAPI co-stained tissue cysts within which the number of nuclei could not be accurately counted was significantly greater at time points associated with high PAS labeling (weeks 5-7). For this reason, measurements relating to the packing density which is dependent in the accuracy of the nuclear count was performed on non-PAS staining tissue cyst from the same cohort at each time point. Damage to nuclei is likely connected to the low pH associated with deposited Schiff periodic acid dye in an AG concentration dependent manner.
